# Cystic Fibrosis Newborn Screening: Progress and Perspectives

**DOI:** 10.3390/ijns12030060

**Published:** 2026-07-29

**Authors:** Marci K. Sontag, Susanna A. McColley

**Affiliations:** 1 Center for Public Health Innovation, Evergreen, CO 80439, USA; smccolley@luriechildrens.org; 2 Stanley Manne Children’s Research Institute, Ann & Robert H. Lurie Children’s Hospital of Chicago, Chicago, IL 60611, USA

Newborn screening (NBS) for cystic fibrosis (CF) was first implemented almost 50 years ago, and many improvements have been made to the initial algorithms, educational approaches, and available therapies. This Special Issue of the *International Journal of Neonatal Screening* highlights the Advances in CF Newborn Screening, from Laboratory Testing to Diagnosis, spanning the breadth of laboratory testing, screening accuracy, challenging diagnoses, and the impact of modulator therapy.

In response to high variation in approaches to CF NBS, the U.S. CF Foundation’s Consensus Guidelines of CF Newborn Screening (Contribution 1) provides recommendations for the optimal implementation of CF NBS. The approach of the committee was to optimize detection of infants with CF, and the guidelines may be seen as aspirational in their approach. To balance this approach, the CF Foundation’s commentary (Contribution 2) reflects on the importance of the guidelines while also noting that the burden on public health programs to implement the guidelines is not insignificant.

Multiple U.S. programs are working to improve CF NBS by addressing components of their algorithms to improve sensitivity, timeliness, and equity. While all programs use immunoreactive trypsinogen (IRT) as the first stage of CF NBS and *CFTR* variant testing as an additional stage, there are numerous approaches to improving sensitivity and specificity around the globe. In this special edition, the California NBS program presents their approach to address challenges with IRT through the calculation of seasonal IRT cutoffs (Contribution 3). The Georgia program presents a comprehensive review of their program from IRT through DNA testing (Contribution 4), and an evaluation of the *CFTR* variant distribution in the clinical population and performance of various panels in identifying those variants (Contribution 5), and Iowa presents the impact that the transition toward improved CFTR testing has had in their program (Contribution 6). As jurisdictions consider the addition of next-generation sequencing in CF NBS, the lessons from Florida’s implementation and subsequent modifications to their approach can help to provide guidance in determining the variants reported and in the referral for the sweat test of infants with one variant following sequencing (Contribution 7).

The complexities of CF newborn screening are also seen after the laboratory testing is completed. The Georgia NBS program has described the importance of follow-up and genetic counseling following a positive NBS (Contribution 8). A consequence of CF NBS is the identification of infants with Cystic Fibrosis Screen Positive Inconclusive Diagnosis/CFTR-Related Metabolic Syndrome (CFSPID/CRMS). The rate of identification of newborns with a CRMS/CFSPID diagnosis varies widely across programs and has increased with the expansion of the *CFTR* variant. Methods for a better understanding of the diagnosis are presented through both a clinical science (Contribution 9) and an epidemiologic approach (Contribution 10).

The international contributions to CF NBS are highlighted from an established NBS program in Portugal, describing their first decade of experience (Contribution 11). However, despite the widespread adoption of NBS for CF in many parts of the world, there are many regions without CF screening. Highlighting this challenge, the need for CF NBS in countries such as India are well described in Contribution 12 and Contribution 13.

The successful implementation of newborn screening relies on good communication in the prenatal and neonatal periods. Neonatologists often receive abnormal CF newborn screening results and may lack knowledge on the next steps in their states (Contribution 14). An emerging challenge in NBS is the interpretation of IRT in infants who have been exposed to modulator therapy in utero, as highlighted in a series of cases reported here (Contribution 15).

The final contribution (Contribution 16) in this special edition provides the capstone of the last 50 years of CF newborn screening. Dr. Phil Farrell shares his unique perspective on the historical landscape that has led CF newborn screening from its infancy to the cutting edge of *CFTR* sequencing. Fittingly, the special edition closes with Dr. Farrell’s predictions for the future of CF NBS, including improved equity, timeliness, and sensitivity, while we reach the goal of improved therapy so that all children with CF may truly live to their full potential.

Progress in CF NBS has been driven by successful collaborations among clinicians, researchers, public health professionals, parents and advocacy organizations around the world. The contributions featured in this special edition reflect the breadth and depth of ongoing efforts to enhance the timeliness, effectiveness, and equity of CF NBS programs. This collection of manuscripts would not have been possible without the dedication of the authors and reviewers, whose work builds upon the foundation established by generations of committed professionals who have advanced the field.

